# Meta-optics empowered vector visual cryptography for high security and rapid decryption

**DOI:** 10.1038/s41467-023-37510-z

**Published:** 2023-04-07

**Authors:** Fei Zhang, Yinghui Guo, Mingbo Pu, Lianwei Chen, Mingfeng Xu, Minghao Liao, Lanting Li, Xiong Li, Xiaoliang Ma, Xiangang Luo

**Affiliations:** 1 State Key Laboratory of Optical Technologies on Nano-Fabrication and Micro-Engineering, Institute of Optics and Electronics, Chinese Academy of Sciences, Chengdu, 610209 China; 2 Research Center on Vector Optical Fields, Institute of Optics and Electronics, Chinese Academy of Sciences, Chengdu, 610209 China; 3 School of Optoelectronics, University of Chinese Academy of Sciences, Beijing, 100049 China; 4 Tianfu Xinglong Lake Laboratory, Chengdu, 610299 China

**Keywords:** Metamaterials, Imaging and sensing

## Abstract

Optical encryption is a promising approach to protecting secret information owing to the advantages of low-power consumption, parallel, high-speed, and multi-dimensional processing capabilities. Nevertheless, conventional strategies generally suffer from bulky system volume, relatively low security level, redundant measurement, and/or requirement of digital decryption algorithms. Here, we propose a general optical security strategy dubbed meta-optics-empowered vector visual cryptography, which fully exploits the abundant degrees of freedom of light as well as the spatial dislocation as key parameters, significantly upgrading the security level. We also demonstrate a decryption meta-camera that can implement the reversal coding procedure for real-time imaging display of hidden information, avoiding redundant measurement and digital post-processing. Our strategy features the merits of a compact footprint, high security, and rapid decryption, which may open an avenue for optical information security and anti-counterfeiting.

## Abstract (web summary)

Usual methods for optical encryption suffer from a tradeoff between the level of security and the complexity of operation given by multiple optical measurements or digital postprocessing. Here, the authors show a multi-d.o.f. metasurface-based vector optical manipulation protocol enabling secure decryption in real time.

## Introduction

Information security is critical for a great number of applications ranging from anti-counterfeiting to telecommunications. Various digital cryptography techniques have been investigated to prevent information leakage, pursuing a high-security level of information. For computer-based techniques, long latency and high computational power are two main challenges. Compared with their electronic counterparts, optical cryptography techniques generally have the advantages of low-power consumption, high-speed parallel processing, and multi-dimensional capabilities, opening up a gate for securing information^1,2^. The past decades have seen significant advances in optical cryptography, including optical watermarking^3^, steganography^4^, and visual cryptography (VC)^5,6^. Nevertheless, the early efforts rely on the complex combination of multiple optical components for signal processing in the Fourier realm^7^, leading to a large form factor. Furthermore, owing to the limited vector optical-field manipulation capabilities of conventional optical devices, the abundant degrees of freedom of light, such as amplitude, phase, frequency, polarization, etc., have not been fully exploited in early optical cryptography, leading to a limited safety performance^8–11^.

In recent years, metasurfaces, one kind of ultrathin optical elements consisting of an array of subwavelength nanostructures, have been developed to manipulate all the fundamental properties of light^12–24^. By combining multiple meta-atoms^25–27^ or different phase shift mechanisms^28–32^, a single metasurface can be engineered to achieve independent multi-dimensional optical-field manipulation^33–35^. The compactness and versatile functionalities make metasurfaces perfect candidates for optical encryption^36–42^, through various mechanisms such as multichannel vector hologram by exploiting Malus’s law^43–45^, the combination of grayscale/color printing and the holographic image^46–50^, as well as tunable meta-holograms based on phase-change materials or spatial light modulators^51–54^. However, the spatially varying polarization property of vector light is not well exploited in optical cryptography until now, leading to a limited security level. The vast majority of holographic cryptography techniques can be potentially cracked by adjusting the polarization state of the input and output light or the incidence wavelength^38,41,44,46^. A pioneering work that combines metasurface with ghost imaging or single-pixel imaging provides a framework to solve the integration problem and enhance the security level^55^. In recent investigations, the security level has been improved by integrating metasurface imaging, visual cryptography, and computational imaging^56,57^. Generally, owing to the indirect imaging manner of computational imaging, multiple optical measurements or additional digital post-processing are required for hidden image restoration. Essentially, these approaches deviate from the original intention of all-optical encryption, leading to the loss of the merits of parallel, high-speed, and low-power consumption properties to some extent.

Here, we propose the concept of high-security vector VC, whose ciphertexts are coded based on the vector imaging process of a spin-decoupled dual-axis metalens. Since the spatial degree is theoretically unlimited and the encryption is combined with other degrees (e.g., incident wavelength, polarization, orbital angular momentum, and spatial dislocation of spin states), our approach enables much higher security. Benefiting from the metasurface-based vector optical manipulation^58^, the complex encryption process can be reversely decrypted via a compact meta-camera. Once these optical key parameters are all matched perfectly, the hidden vector optical information is converted into detectable intensity patterns, enabling secure decryption in real-time without additional measurement and digital post-processing. Owing to the advantages of a compact footprint, high-security level, and real-time security display (see Supplementary Note 1 for a comprehensive comparison among different optical cryptography techniques), the proposed vector VC is favorable for optical information security and anti-counterfeiting. These properties are of significance in the future development of optical security^1,59,60^, especially in monitored/peeped environments^5,61^.

## Results

### Concept and operation principle of the decryption camera

**Fig. 1:**
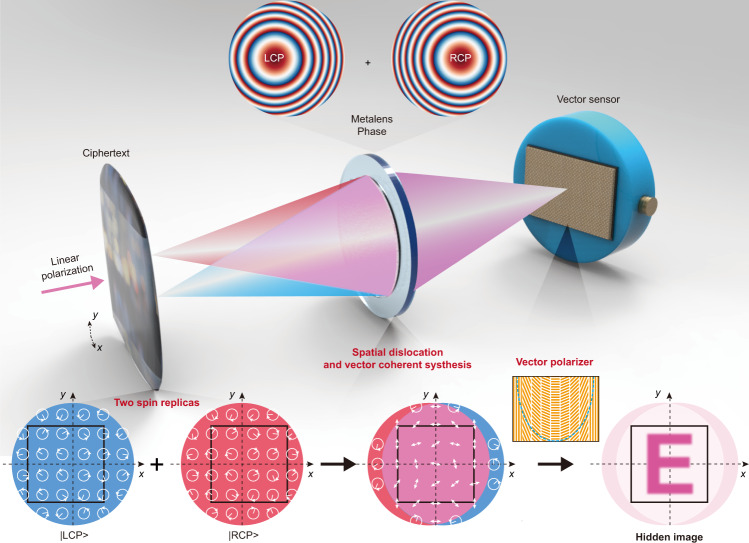
Schematics of a meta-optics-empowered decryption camera for vector VC. The decryption camera is composed of spin-decoupled dual-axis metalens (the phase profile is schematically shown in the top panel) and a vector polarization-analyzer sensor (indicated in the inset). The spin-decoupled dual-axis metalens divides the coded phase-type ciphertexts into two replicas with opposite circular polarized states and projects them to the image plane with spatial dislocation and overlap (bottom panel). The handedness and phase at each pixel are represented by the direction of the circle arrows and the azimuthal angle of short wires, respectively. As a reversal process of the coding procedure, the vector optical field synthesized at the spatially overlapping region on the image plane reproduces the hidden image in the optical ciphertexts with the help of a matched vector polarization analyzer.

Most conventional VC devices decompose secret images into multiple binary amplitude or phase shares, which are then digitally or physically overlapped to recover the original secret image. Compared with existing intensity- and phase-only scalar VC, our proposed vector VC exhibits a higher security level since it fully exploits abundant degrees of freedom of light as key parameters, including wavelength, phase, amplitude, polarization, and spatial dislocation. Therefore, the hidden image in a ciphertext cannot be directly decrypted by simply rotating the polarization state of the input and output light^54^, ensuring a high-security level. To decrypt the optical ciphertext images, we construct a user-defined meta-camera based on the vector imaging principle, which could realize real-time decryption without complex mathematical operations or additional computational hardware resources. As indicated in Fig. 1, the decryption meta-camera contains two unique decryption elements: a) spin-decoupled dual-axis metalens generating pixel-wise spatial dislocation and overlap between two spin replicas of a ciphertext image and b) a vector sensor comprising a vector polarization analyzer attached to a photodetector for rapid security display.

Specifically, the spin-decoupled metalens equivalently operates as two independent devices with full-aperture utilization, owing to the conjugate symmetry breaking of photonic spin-orbit interactions via combining both propagation and geometric phases in a single metasurface^25,28^. Here, the spin-decoupled metalens is designed as a dual-axis planar lens to respectively respond to the left-handed and right-handed circularly polarized (LCP and RCP) light coming from the optical ciphertext. Consequently, the single-to-single point mapping of a conventional lens is transformed into dual-to-single or single-to-dual mapping. As shown in the bottom of Fig. 1, when an optical ciphertext is placed at a proper distance from the spin-decoupled metalens, it divides the optical ciphertext into two replicas with opposite spin states and projects them to the image plane with a certain spatial dislocation and overlap. The optical ciphertext exhibits a spatially inhomogeneous phase, resulting in the inherent phase difference between two points of the ciphertext. In addition, there will be a spatial variation between the propagation phases accumulated along distinct optical paths, when two separated pixels on the ciphertext respectively pass through the dual-axis centers of the metalens and intersect at one point on the image plane. Furthermore, the linear polarization direction of incidence will introduce a uniform phase difference between the two spin replicas. Owing to the inhomogeneous distribution (phase, amplitude, or both) of two spin replicas caused by the aforementioned factors, a complex vector optical field is generated in their overlapping region at the image plane. For ease of understanding, Fig. 1 shows a case of the phase-type ciphertext. Since conventional cameras cannot directly characterize the distribution of vector light fields, another vital decryption element called vector sensor is constructed by tightly attaching a vector polarization analyzer to a normal photodetector. The vector polarization analyzer is composed of meta-gratings with spatially varying orientations. It converts the synthesized vector optical field into intensity patterns, wherein the intensity value is determined by both the aforementioned phase difference and the orientations of meta-gratings.

**Fig. 2:**
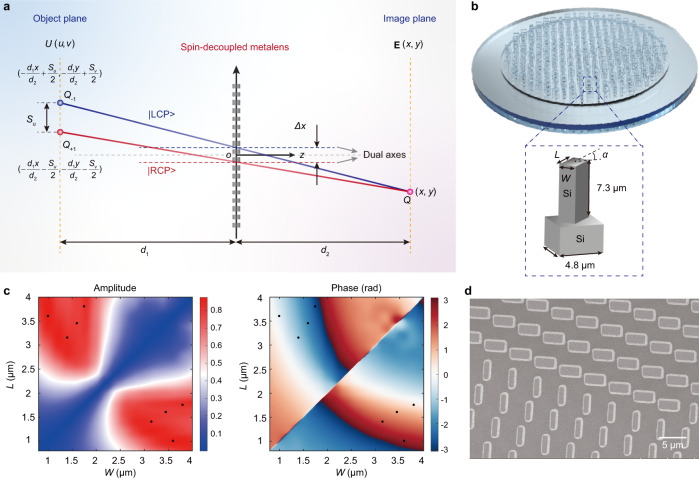
Spatial mapping relationship of the spin-decoupled metalens. **a** Opposite spin components of pixels *Q*_+1_ and *Q*_−1_ at the object plane are imaged by the spin-decoupled dual-axis metalens and coherently synthesized at the pixel *Q* on the image plane. **b** Schematics of the spin-decoupled metalens with the nanopillar building block shown in the inset. The width *W*, length *L*, and spatial orientation *α* are spatially engineered according to the phase profiles of LCP and RCP. **c** Polarization conversion amplitude and the phase shift as functions of *W* and *L*. The black dots represent the selected eight unit cells with high conversion efficiency and equal phase step. **d** Scanning electron microscope (SEM) images of the metalens consisting of all-silicon nanopillars with different geometries and orientations.

The imaging mechanism of our decryption camera is equivalent to the reversal process of the coding procedure. As shown in Fig. 2a, under the illumination of linearly polarized light (composed of two spin states represented by *σ* = ±1), any pixel (denoted as *Q*) located at the overlapping region on the image plane is the vector coherent synthesis of RCP and LCP components coming from two separated points (denoted as *Q*_+1_ and *Q*_−1_) on the object plane via the spin-decoupled dual-axis metalens. The design of the spin-decoupled metalens is shown in Supplementary Note 2. Without loss of generality, we assume that the camera operates with a distortion-free magnification factor of *d*_2_/*d*_1_, where *d*_1_ and *d*_2_ are the object and image distances, respectively. If the pixel size of the ciphertext is sufficiently larger than the detection resolution, the image can be approximated to a point-to-point projection from (*u*, *v*) to (*x*, *y*) (the diffraction effect along the optical path is ignored). Under a circularly polarized basis, the output electric fields distribution can be approximately written as:

1 $$	{{{\mathbf E}}}(x,y)\propto \mathop{\sum}\limits_{\sigma }{e}^{i(k\overline{{Q}_{\sigma }Q}-\sigma \theta )}U(u,v)\left[\begin{array}{c}1\\ -\sigma i\end{array}\right]\\ 	=\mathop{\sum}\limits_{\sigma }{e}^{i(k\overline{{Q}_{\sigma }Q}-\sigma \theta )}U\left(-\frac{{d}_{1}x}{{d}_{2}}-\frac{\sigma {S}_{u}}{2},-\frac{{d}_{1}y}{{d}_{2}}-\frac{\sigma {S}_{v}}{2}\right)\left[\begin{array}{c}1\\ -\sigma i\end{array}\right],$$

and

2 $$k=\frac{2\pi }{\lambda },{S}_{u}=\varDelta x\left(\frac{{d}_{1}}{{d}_{2}}+1\right),{S}_{v}=\varDelta y\left(\frac{{d}_{1}}{{d}_{2}}+1\right),$$

where $k\overline{{Q}_{\sigma }Q}$ represents the propagation phase along the optical path, (Δ*x*, Δ*y*) is the lateral displacement between the two axes at the metalens plane, (*S*_*u*_, *S*_*v*_) is the lateral displacement between points *Q*_+1_ and *Q*_−1_, and *λ* indicates the wavelength. Furthermore, −*σθ* represents the polarization-dependent phase delay of two spin components for a linearly polarized incidence with an azimuthal angle *θ*, while $U(u,v)=A(u,v)\exp [i\varphi (u,v)]$ is the complex-amplitude distribution function of the ciphertext (*A* is the amplitude and *φ* is the phase). There are three phase factors in the right-hand of Eq. (1) contributing to the phase differences between two spin replicas, which respectively depend on the different propagation phase, the azimuthal angle of the incident polarization, and the inherent phase of the ciphertext.

When the size of the ciphertext is much smaller than the focal length, the paraxial approximation holds and **E**(*x*,*y*) can be approximately simplified with the spin-independent phase being ignored:

3 $${{{{{\bf{E}}}}}}(x,y)\approx \mathop{\sum}\limits_{\sigma }{e}^{i[\sigma \chi (x,y)-\sigma \theta ]}U\left(-\frac{{d}_{1}x}{{d}_{2}}-\frac{\sigma {S}_{u}}{2},-\frac{{d}_{1}y}{{d}_{2}}-\frac{\sigma {S}_{v}}{2}\right)\left[\begin{array}{c}1\\ -\sigma i\end{array}\right],$$

where *χ* indicates the halved propagation phase difference between two distinct optical paths and can be written as:

4 $$\chi (x,y)=k\frac{x{S}_{u}+y{S}_{v}}{2{d}_{2}}=\frac{k(x\cdot \varDelta x+y\cdot \varDelta y)({d}_{1}+{d}_{2})}{2{{d}_{2}}^{2}}.$$

Since the complex-amplitude of the ciphertext and the optical-path-determined propagation phase difference are changed among the spatial pixels, the final polarization distribution is inhomogeneous and complex, as illustrated in Supplementary Note 3. Since the polarization states of the two spin replicas are orthogonal, there will be no interference-induced intensity change after synthesis. To address this issue, a vector linear polarization analyzer with a spatially varying orientation of *γ*(*x*, *y*) is required at the front of sensors to form the complex interference effect between the two dislocated replicas and then convert the phase difference and amplitude into detectable intensity patterns, which can be written as:

5 $$I(x,y)={ \left | \mathop{\sum}\limits_{\sigma }{e}^{i\sigma [\chi (x,y)-\theta -\gamma (x,y)]}U\left(-\frac{{d}_{1}x}{{d}_{2}}-\frac{\sigma {S}_{u}}{2},-\frac{{d}_{1}y}{{d}_{2}}-\frac{\sigma {S}_{v}}{2}\right) \right | }^{2}.$$

In principle, the more complex the orientation distribution of *γ*, the higher the security. For simplicity, we utilize a catenary-like vector polarization analyzer with its spatial orientation $\gamma (x,y)=\chi (x,y)-\theta+\pi /2$, as discussed in Supplementary Note 4. Since its orientation profile linearly changes with the coordinates, its structural streamline, illustrated as the blue dash curve in Fig. 1, forms the catenary of equal strength^14^. Under this condition, the final intensity pattern through the vector polarization analyzer is obtained:

6 $$I(x,y)={ \left | \mathop{\sum}\limits_{\sigma }\sigma U\left(-\frac{{d}_{1}x}{{d}_{2}}-\frac{\sigma {S}_{u}}{2},-\frac{{d}_{1}y}{{d}_{2}}-\frac{\sigma {S}_{v}}{2}\right) \right | }^{2}.$$

As illustrated by Eq. (4–6), the imaging result of our decryption meta-camera is the interference result between different spatial pixels in the ciphertext, accompanied by a phase difference of π. The final intensity distribution is determined by several critical key parameters (*λ*, Δ*x*, Δ*y*, *d*_1_, *d*_2_, *θ*, and *γ*). The abundance of key parameters ensures a high-security level of the complex-amplitude vector VC. Therefore, it is unlikely to decrypt the vector VC by try-and-error attack within a reasonable time if these key parameters are unknown.

Subsequently, we show how to encrypt an arbitrary hidden intensity image *I* into the ciphertext *U*. Without loss of generality and for simplicity, it is assumed that $\varDelta y=0$, *I* has a pixel number of ${q}_{1}\times {q}_{2}$ with a pixel size of $\varDelta x\times \varDelta x$, and ${S}_{u}=n\varDelta x$, where *n* is a positive integer. To realize dislocation encryption between different columns, *U* requires at least ${q}_{1}\times ({q}_{2}+n)$ pixels for complete image restoration. The first *n* columns of *U* are random complex numbers, therefore, the same hidden image *I* can be encrypted into many different ciphertexts. An example of the same information hidden in multiple different ciphertexts is presented in Supplementary Note 5.

In general, the *l*th row and *m*th column of *U* is given by:

7 $$U(l,m)=\left\{\begin{array}{c}U(l,m-n)-\sqrt{I(l,m-n)}{e}^{i{\psi }_{1}}\\ or\\ U(l,m-n)+\sqrt{I(l,m-n)}{e}^{i{\psi }_{1}}\end{array}\right.,\;m \, > \,n\ge 1$$

where the one with the smaller modulus is selected. For *m* ≤ *n*, there are:

8 $$U(l,m)=a{e}^{i{\psi }_{2}}$$

Here, *ψ*_1_ and *ψ*_2_ are random phases ranging from 0 to 2π and *a* is a random number ranging from 0 to 1. In our design, *ψ*_1_ is equal to zero for simplicity. These random codes in the ciphertexts (the first *n* columns of *U*) and hidden image (its phase profile) can reduce the probability that directly inferring the hidden image *I* from the complex amplitude of the ciphertext.

### Experimental demonstration and security verification of the vector VC

In the decryption meta-camera, the phase coefficients and profiles of the spin-decoupled metalens for RCP and LCP components are given in Supplementary Note 2. The distinct phase profiles of LCP and RCP are implemented by merging the propagation phase (*β*) and geometric phase (−2*σα*) in a single metasurface, which is composed of high-index dielectric rectangular nanopillars with various geometries and orientations (*α*). Among them, the propagation phase is spin-insensitive and utilized to generate the focusing wavefront, while the spin-dependent geometric phase introduces an opposite linear phase gradient to the focusing wavefront, leading to the generation of the dual-axis metalens. These phases can be independently controlled by the spatial geometries and orientations of the nanopillar. Under the normal illumination of circularly polarized light of ${[1,-\sigma i]}^{{{{{{\rm{T}}}}}}}$, the output field from the nanopillars follows an analytical model based on the Jones matrix given by (see Supplementary Note 2 for details)^28,31^:

9 $${{{{{{\bf{E}}}}}}}_{atom}=\,\cos \frac{\delta }{2}{e}^{i\beta }\left[\begin{array}{c}1\\ -\sigma i\end{array}\right]+i\,\sin \frac{\delta }{2}{e}^{i(-2\sigma \alpha+\beta )}\left[\begin{array}{c}1\\ \sigma i\end{array}\right],$$

where *β* + *δ*/2 and *β-δ*/2, respectively, denote the phase shifts along the two main axes of the nanopillar and *α* is its orientation angle. The first term in Eq. (9) is determined by the propagation phase and shares the same polarization with the incidence. In contrast, the second term reverses the handedness of the incidence and imparts not only the geometric phase but also the propagation phase. These phases can be independently controlled by the spatial orientation and geometries of the nanopillars, which are required in constructing the spin-decoupled metalens^25,28^. Note that the phase difference of *δ* between two anisotropic axes generally equals to π for high energy efficiency.

For proof-of-concept demonstrations, the vector VC is demonstrated at the wavelength of 10.6 μm. We utilized all-silicon nanopillars with high transmissivity at this wavelength as meta-atoms, which are arranged in a square lattice with the period and height being kept (*p*_*x*_ = *p*_*y*_ = 4.8 µm and *h* = 7.3 µm), as indicated in Fig. 2b. The amplitude and phase of the spin-reversed light as functions of width *W* and length *L* are determined through the finite-element method and the simulation results are presented in Fig. 2c. As indicated by the black points in Fig. 2c, a set of eight nanopillars with proper *W* and *L* (the geometries are listed in Supplementary Table 3), including eight basic structures are utilized to provide eight phase levels covering the 2π phase range with high average polarization conversion amplitude (higher than 0.8). Then, the geometries and spatial orientation of the meta-atoms at each pixel can be determined by the required phase profiles for RCP and LCP (${\zeta }_{+1}$ and ${\zeta }_{-1}$)^25,28^. The designed metalens are fabricated by direct laser writing (see Methods for details), and the SEM image is presented in Fig. 2d, demonstrating high fabrication quality. Note that, our vector VC can be extended to the near-infrared and the visible band. The direct laser writing can be replaced by electron-beam lithography for smaller feature fabrication. The spin-decoupled metalens and corresponding meta-atoms operating at the communication band of 1.55 μm are presented in Supplementary Note 2.

**Fig. 3:**
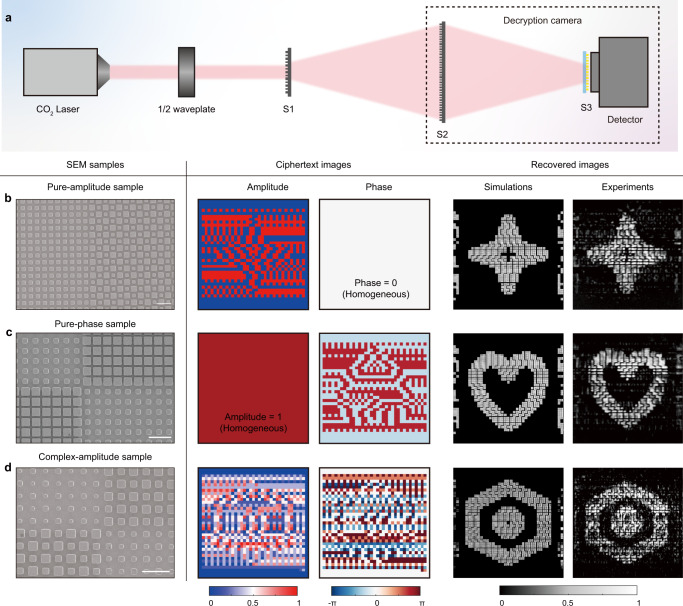
Experimental setup and decryption results. **a** Experimental setup. S1 to S3 represent the optical ciphertext, spin-multiplexed metalens, and the vector polarizer, respectively. **b–d** Simulated and experimental imaging results of three kinds of optical ciphertexts with binary pure-amplitude, binary pure-phase, or grayscale complex-amplitude modulation. Left panel: SEM images of the optical ciphertexts. Middle panel: complex-amplitude distributions of the optical ciphertexts. Right panel: Simulated and measured imaging results. Scale bar: 10 μm.

Figure 3a shows our experimental setup. A proper incident linear polarization is critical to illuminate the steganographic ciphertext (S1), which serves as the first key in the decryption process. S2 and S3 represent the spin-decoupled metalens and vector polarization analyzer, respectively. The imaging system parameters are summarized as follows: *d*_1_ = *d*_2_ = 130 mm (the focal length is 65 mm), Δ*x* = 100 μm, Δ*y* = 0, *θ* = 0, and *S*_*u*_ = 2Δ*x* (i.e., *n* in Eq. (7) is equal to 2). The simulation results shown in Supplementary Note 4 indicate our vector polarizer supports a high extinction ratio of ~1000:1 and transmissivity of ~85%, which can be further improved by decreasing the period and structural height. To demonstrate the robustness of our system, three kinds of optical ciphertexts with binary pure-amplitude (Fig. 3b), binary pure-phase (Fig. 3c), and grayscale complex-amplitude (Fig. 3d) encryption are fabricated and characterized. Multiple meta-atom interference approach proposed in our previous work^25^ is employed to generate such an optical ciphertext with arbitrary complex-amplitude distributions *U* (see Supplementary Note 6 for details), which can be directly decrypted by the proposed decryption meta-camera with proper design and a set of matched key parameters. The SEM images, complex-amplitude distributions of these optical ciphertexts, and simulated and measured imaging results are shown in the left, middle, and right panels of Fig. 3b–d. The measured results are consistent with simulation results, which indicates the encrypted information can be effectively retrieved by our system due to its capability to detect and analyze vector optical fields. Supplementary Movie 1 shows a rapid decryption process.

**Fig. 4:**
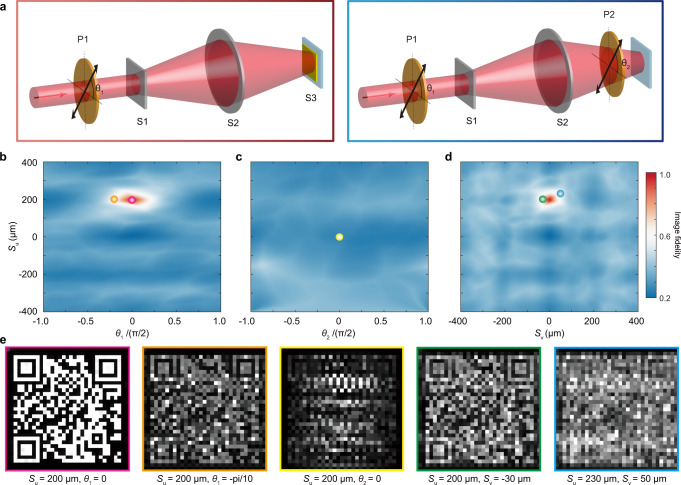
Security verification of the vector VC. **a** Schematic of optical setups for sensitivity check. Left panel: changing the incident polarization direction by rotating a normal linear polarizer 1 (P1). Right panel: changing the detection polarization direction by rotating a normal linear polarizer 2 (P2) that replaces the vector polarization analyzer (S3). Two-dimensional map of image fidelity as a function of the horizontal dislocation of *S*_*u*_ and incident polarization angle of *θ*_1_ (**b**), detection polarization angle of *θ*_2_ (**c**), or vertical dislocation of *S*_*v*_ (**d**). **e** Simulated imaging results of a hidden QR code at different key parameters, represented by circles of different colors in (**b–d**).

As discussed above, the optical decryption requires a specially equipped meta-camera with proper designs and key parameters: i.e., a spin-decoupled dual-axis metalens with matched key parameters (*S*_*u*_ and *S*_*v*_ determined by Δ*x*, Δ*y*, *d*_1_, and *d*_2_), a vector polarization analyzer with a matched spatial orientation $\gamma=\chi -\theta+\pi /2$, and a correct incident polarization state (linear polarization with an azimuthal angle of *θ*). Missing any of these secret keys will result in a failed decryption. The following simulations are carried out for security verification of the vector VC under different physical parameters. Figure 4a displays schematic diagrams of optical setups for characterizing the sensitivity of image fidelity to the incident and detection polarization directions, which are controlled by rotating two normal linear polarizers P1 and P2, respectively. The vector polarization analyzer (S3) is utilized in the left panel case but is replaced by the linear polarizer (P2) in the right panel case. The image fidelity is defined as $1-\sum|\overline{I}-{\overline{I}}_{o}|/\sum|\overline{I}+{\overline{I}}_{o}|$ to measure the similarity between the retrieved image and hidden image, where $\overline{I}$ and ${\overline{I}}_{o}$, respectively represent the normalized intensity of the measured pattern and hidden pattern. Figure 4b–d shows the two-dimensional map of image fidelity as a function of the horizontal dislocation of *S*_*u*_ and incident polarization angle of *θ*_1_, detection polarization angle of *θ*_2_, and vertical dislocation of *S*_v_. It can be seen that confidential information can only be retrieved with relatively high image fidelity in a small area, where the key parameters exactly approach the designed system parameters. Note that, the actual selectable parameter space is larger than the simulation range. For example, dislocation parameters of *S*_*u*_ and *S*_*v*_ can go well beyond ±400 μm, and both incident and detection polarizations can be at any point on the Poincaré sphere (e.g., security verifications under circularly polarized incidences are presented in Supplementary Note 7). Figure 4e shows the simulated imaging results of a hidden QR code at different key parameters, represented by circles of different colors in Fig. 4b~d. We can see the hidden QR code can be reconstructed precisely at the center of the hot spots.

To retrieve the hidden image, a matched vector polarization analyzer is necessary but insufficient. Especially when the vector polarization analyzer (S3) is replaced by a normal linear-polarization analyzer (P2) in the camera, the image fidelity is always low. In other words, confidential information cannot be obtained, no matter what the incidence polarization is. If other parameters have been known by the attacker and the hidden imaging is relatively simple, normal polarization filters jointly with physics-driven machine-learning modeling could extract relevant information through multiple measurements. However, this potential attack can be readily reduced by using a hybrid vector polarization analyzer during the encoding of ciphertexts. For example, the catenary-like vector linear polarization analyzer could be replaced by a full-Poincaré vector polarization analyzer based on the local interference principle^25,27^.

## Discussion

To summarize, we present a decryption meta-camera and a general approach for real-time extraction of hidden images in coded complex-amplitude ciphertexts. Three kinds of optical ciphertexts with binary pure-amplitude, binary pure-phase, and grayscale complex-amplitude encryption are designed and fabricated to demonstrate the flexibility of this method for vector VC. Furthermore, the high-security level of the meta-optics-based vector VC is examined, which simultaneously requires a proper incidence polarization, a correct imaging configuration, and a matched vector polarization analyzer. Inspired by the spatial vector coding empowered by spin-decoupled metasurfaces^62,63^, we expect the meta-optics-based camera to obtain all-optical sensing-computing capability in the near future, which combines the sensing and computing in a single meta-device without any electronic post-processing. The capabilities of parallel high-speed processing and multi-dimensional manipulation may open an avenue for vector optical field research as well as intelligent perception and recognition.

The proposed vector VC has good scalability to further enhance its security without the sacrifice of responding time and convenience. For example, the incident polarization state can be coded to apply to a special elliptical polarization or even a vector field. The cryptography complexity can also be increased by using hybrid vector depolarizers or extending the dual-axis metalens to multi-axis metalenses (e.g., each circular polarization corresponds to multiple focus points enabled by complex-amplitude superposition^64^) for complicated spatial dislocation among multiple replicas. Furthermore, owing to the advantage of rapid decryption, one can design meta-ciphertexts containing volatile materials to maintain the correct complex amplitude in a given condition, while the device can be destroyed under unauthorized try-and-error attack. In addition, combined with the recent advance in spatial nonlinear optics^10^, one can develop a nonlinear vector VC encryption system to reduce the risks caused by the fixed linear relationship between the ciphertext and hidden image, including known-plaintext attack, chosen-plaintext attack, and deep-learning-based attack.

## Methods

The metalens and ciphertexts were fabricated through direct laser writing (Heidelberg DWL66+). The minimum feature size is about 0.6 μm, which is smaller than the geometries of the meta-atoms. First, ~700 nm thick photoresist is spin-coated on a double-sided polished silicon wafer with a thickness of 0.5 mm. An inductively coupled plasma etching combined with the Bosch process was subsequently employed to transfer photoresist patterns into the silicon wafer to form silicon structures. For the vector polarization analyzer, a double-sided polished barium fluoride wafer was deposited with a 600-nm gold layer using magnetron sputtering. After laser direct writing, ion beam etching was applied to transfer photoresist patterns into gold patterns, and the remaining photoresist was removed by reactive ion etching.

## Supplementary information

Supplementary Information

Description of Additional Supplementary Files

Supplementary Movie 1

## Data Availability

The source data are available from the corresponding author upon request. All data needed to evaluate the conclusion are present in the manuscript and/or the Supplementary Information.
