# Supplementary material for: Meta-optics empowered vector visual cryptography for high security and rapid decryption
Source: Nat Commun. 2023 Apr 7;14:1946. doi: 10.1038/s41467-023-37510-z (PMC10081998; doi:10.1038/s41467-023-37510-z)
Supplement: Supplementary file 1 — Supplementary Information [file 41467_2023_37510_MOESM1_ESM.pdf]

Supplementary Information for

Meta-optics empowered vector visual cryptography for high security and rapid decryption

Fei Zhang[1,2†], Yinghui Guo[1,2,3†], Mingbo Pu[1,2,3*], Lianwei Chen[1], Mingfeng Xu[1,2], Minghao Liao[1,3], Lanting Li[4], Xiong Li[1,3], Xiaoliang Ma[1,3], and Xiangang Luo[1,3*]

[1]State Key Laboratory of Optical Technologies on Nano-Fabrication and Micro-Engineering, Institute of Optics and Electronics, Chinese Academy of Sciences, Chengdu 610209, China.

[2]Research Center on Vector Optical Fields, Institute of Optics and Electronics, Chinese Academy of Sciences, Chengdu 610209, China.

[3]School of Optoelectronics, University of Chinese Academy of Sciences, Beijing 100049, China.

[4]Tianfu Xinglong Lake Laboratory, Chengdu 610299, China.

†These authors contributed equally to this work.

*Email: pmb@ioe.ac.cn, lxg@ioe.ac.cn

Supplementary Note 1: Comparisons among different optical cryptography techniques

Supplementary Note 2: Design and implementation of the spin-decoupled metalens

Supplementary Note 3: Vector coherent synthesis of two spin replicas at the image plane

Supplementary Note 4: Design of the vector polarization analyzer

Supplementary Note 5: Encrypt one confidential image into distinct ciphertexts

Supplementary Note 6: Design of the complex-amplitude ciphertext

Supplementary Note 7: Security verification under circularly polarized incidence

Supplementary Note 1: Comparisons among different optical cryptography techniques

Optical encryption is a promising approach to protecting secret information, but conventional strategies generally suffer from bulky system volume, relatively low-security level, redundant measurement, and/or requirement of digital decryption algorithms. Until now, the spatially varied polarization property of vector light is not fully exploited in optical cryptography, leading to a limited security level. Therefore, the vast majority of meta-optics-based cryptography techniques[1–7] can be potentially cracked by adjusting the polarization state of the input and output light or the illumination wavelength. Conventional approaches to enhance the security level of meta-optics-based cryptography techniques are combining holographic cryptography with computational imaging[8–10], e.g. ghost imaging or single-pixel imaging. However, computational imaging is an indirect imaging manner, which requires tremendous measurements and complex digital post-processing for image restoration, significantly impeding the decryption speed and measurement convenience. Essentially, these approaches deviate from the original intention of all-optical encryption, and thus cancel the merits of low-power consumption, parallel, high-speed, and multi-dimensional processing capabilities to some extent. Hence, the main bottleneck problem in this realm is the fact that high security and rapid decryption speed can hardly be achieved simultaneously.

To further highlight the novelty and impact, we have made a comprehensive comparison among different optical cryptography techniques in system compactness, security level, decryption speed, and measurement convenience.

Supplementary Table 1. Comparisons among different optical cryptography techniques in system compactness, security level, real-time decryption, and measurement convenience.

**Optical cryptography techniques**	**System compactness**	**Security level**	**Real-time decryption**	**Measurement convenience**
Double random phase encoding[11–14]	★[a]	★★★	★★★	★★[b]
Amplitude- or phase-only scalar VC[15–18]	★★★	★[c]	★★★	★★[d]
Polarization encryption holography[1–5]	★★★	★[e]	★★★	★★★
Holography combined with computational imaging[8–10]	★★★	★★★	★[f]	★★[g]
**Meta-optics-based vector VC**	★★★	★★★	★★★	★★★

a) Typically require a 4-*f* system to perform the transformation between spatial and Fourier realm;
b) The decryption process typically requires a reference light;
c) The encryption schematic is simple via tailoring the spatial overlapping;
d) Typically require to find a precise alignment between two secrete shares;
e) The ciphertext can be potentially decrypted by rotating the direction of a polarizer or a waveplate;
f) The decryption typically requires additional time and resources for image post-processing;
g) The decryption typically requires multiple measurements and image post-processing.

Supplementary Note 2: Design and implementation of the spin-decoupled metalens

Before introducing the design of the spin-decoupled metalens and its phase profile, a single-axis planar lens is optimized first to ensure good imaging quality. Supplementary Fig. 1a shows the optical layout of a planar lens phase profile optimized based on the ray tracing method in Zemax with the form given by:

$$\zeta(x,y)=\sum_j a_j(x^2+y^2)^j \tag{1}$$

where a_j (j = 1 to 5) are the phase coefficients of the polynomial. Then, for a spin-decoupled metalens with a lateral displacement (Δx, Δy) between its dual-axis, the phase profiles of left-handed and right-handed circularly polarized (LCP and RCP, $\sigma = \pm 1$ indicate spin states) can be written as:

$$\begin{aligned}\zeta_\sigma(x,y)&=\zeta(x+\frac{\sigma\Delta x}{2},y+\frac{\sigma\Delta y}{2})\\&=\sum_j a_j[(x+\frac{\sigma\Delta x}{2})^2+(y+\frac{\sigma\Delta y}{2})^2]^j\end{aligned} \tag{2}$$

Specifically, for an all-silicon planar lens operating at the wavelength of 10.6 μm with a thickness of 0.5 mm and diameter of 5 cm, when the object and image distances are set as $d_1 = d_2$ = 13 cm, the phase profile is present in Supplementary Fig. 1b with the values of a_j listed in Supplementary Table 2. As can be seen from Supplementary Fig. 1c, all light rays are converged within the Airy disk for different image heights. Furthermore, their modulation transfer functions (MTF) are very close to the diffraction limit, as shown in Supplementary Fig. 1d.

To realize the phase profile expressed in Supplementary Eq. (2), the theory of composite phase engineering is employed[19–21]. Without loss of generality, we assume a transparent anisotropic nanopillar whose phase shifts along two axes are $\beta+\delta/2$ and $\beta-\delta/2$. By rotating the nanopillar by an angle of α, its Jones Matrix in Cartesian basis can be written as:

$$\mathbf{H}=\begin{bmatrix}\cos\alpha & -\sin\alpha\\ \sin\alpha & \cos\alpha\end{bmatrix}\begin{bmatrix}e^{i(\beta+\delta/2)} & 0\\ 0 & e^{i(\beta-\delta/2)}\end{bmatrix}\begin{bmatrix}\cos\alpha & \sin\alpha\\ -\sin\alpha & \cos\alpha\end{bmatrix} \tag{3}$$

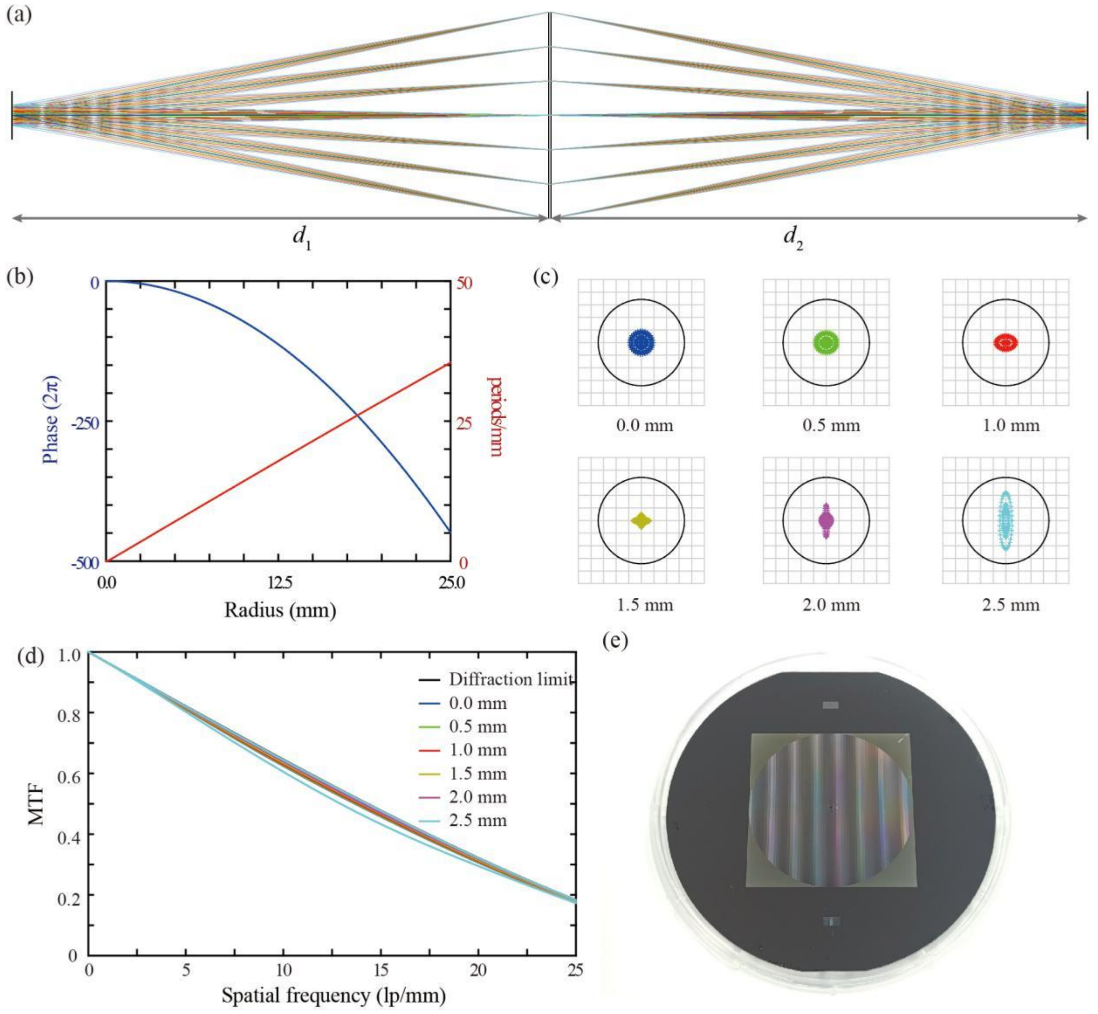

Supplementary Fig. 1. Imaging performance of a planar lens at the wavelength of 10.6 μm. (a) Optical layout of the imaging system with the planar lens. (b) Phase profile of the planar lens. (c) Spot diagrams for different image heights. (d) Optical MTF for different image heights. (e) Photography of the fabricated spin-decoupled metalens.

Supplementary Table 2. Phase coefficients of the planar lens at the 10.6 μm wavelength.

a_1	a_2	a_3	a_4	a_5
-4.5595113	9.5011876×10^{-5}	-9.5799597×10^{-8}	1.4231544×10^{-10}	-7.9701476×10^{-14}

Under the normal illumination of the circularly polarized light $[1, -\sigma i]^{\mathrm{T}}$, the output light through the nanopillars can be given as:

$$\mathbf{E} = \cos\frac{\delta}{2}e^{i\beta}\begin{bmatrix}1\\ -\sigma i\end{bmatrix} + i\sin\frac{\delta}{2}e^{i(-2\sigma\alpha+\beta)}\begin{bmatrix}1\\ \sigma i\end{bmatrix} \qquad (4)$$

As can be seen from Supplementary Eq. (4), the first term shares the same polarization with the incidence but only picks the propagation phase. In contrast, the

second term reverses the handedness of the incidence and imparts not only the geometric phase of -2$\sigma\alpha$ but also the propagation phase of β. These phases can be independently controlled by the spatial orientation and geometries of the nanopillar, which is exactly needed in constructing the spin-decoupled metalens. Therefore, the phase difference of δ between two anisotropic axes generally equals π for high energy efficiency. Then, the geometries and spatial orientation of the meta-atoms at each pixel can be determined by the required phase profiles for RCP and LCP (ζ_{+1} and ζ_{-1}):

$$\begin{aligned}\beta &= [\zeta_{+1} + \zeta_{-1}]/2 \\ \alpha &= [\zeta_{+1} - \zeta_{-1}]/4\end{aligned}. \tag{5}$$

We utilize all-silicon nanopillars with high transmissivity at 10.6 μm as meta-atoms, which are arranged in a square lattice with the constant period and height ($p_x = p_y$ = 4.8 μm and h = 7.3 μm), as indicated in Fig. 2b. The amplitude and phase of the spin-reversed light as functions of width W and length L are determined through the finite-element method and the simulation results are presented in Fig. 2c. As indicated by the black points in Fig. 2c, a set of eight nanopillars with the proper selection of W and L (the geometries are listed in Supplementary Table 3) are utilized to provide eight phase levels covering the 2π phase range with high average polarization conversion amplitude (>0.8). The photography of the fabricated spin-decoupled metalens is shown in Supplementary Fig. 1e.

Supplementary Table 3. Geometries of selected eight nanopillars at 10.6 μm wavelength and simulated polarization conversion amplitude.

	ID1	ID2	ID3	ID4	ID5	ID6	ID7	ID8
Width (μm)	3.8	3.45	3.15	3.6	1.75	1.6	1.4	1.0
Length (μm)	1.75	1.6	1.4	1.0	3.8	3.45	3.15	3.6
Amplitude	0.81	0.78	0.82	0.86	0.81	0.78	0.82	0.86

Note that, such a setup and metalens can be scaled to the near-infrared and the visible band, where the direct laser writing is replaced by electron-beam lithography for smaller feature fabrication. The meta-atoms constructing the spin-decoupled

metalens operating at the telecom band of 1.55 μm are presented here. In this respect, both object and image distances are scaled to 13 mm. The diameter of the metalens is 5 mm. The substrate of the metalens is set as sapphire (Al_2O_3) and has a thickness of 0.2 mm. The phase coefficients are displayed in Supplementary Table 4 and the phase profile is plotted in Supplementary Fig. 2a. Furthermore, the MTF for different image heights are very close to the diffraction limit, as shown in Supplementary Fig. 2b.

Supplementary Table 4. Phase coefficients of the metalens at the 1.55 μm wavelength.

a_1	a_2	a_3	a_4	a_5
-311.8318	3.0065198	-1.2896542	0.25300641	-0.01707753

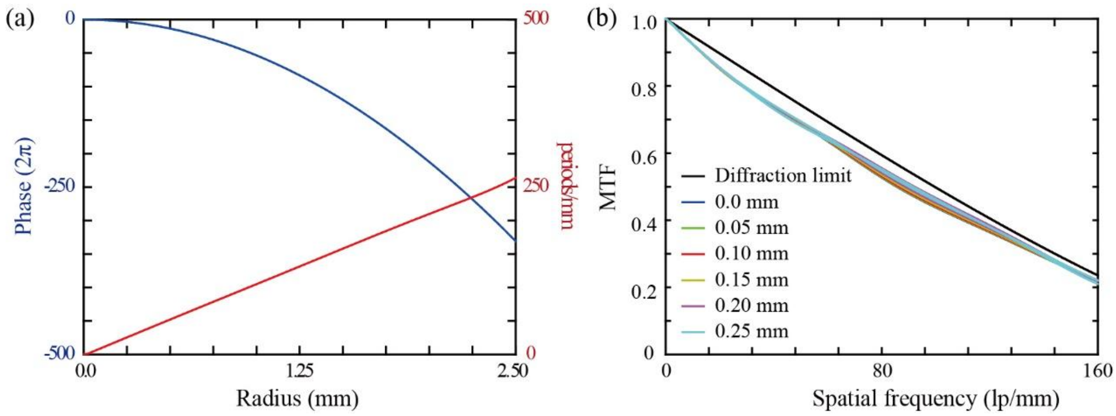

Supplementary Fig. 2. Imaging performance of the planar lens at the wavelength of 1.55 μm. (a) Phase profile of the planar lens. (b) Optical MTF for different image heights.

The meta-atom operating at the wavelength of 1.55 μm is shown in Supplementary Fig. 3a, which is arranged in a square lattice with the constant period and height ($p_x = p_y = 630$ nm and $h = 900$ nm). The materials of the nanopillar and substrate are silicon (Si) and sapphire (Al_2O_3), respectively. We also select eight unit-cells for composite phase modulation, whose geometries are listed in Supplementary Table 5 and the corresponding polarization conversion amplitude and propagation phase are shown in Supplementary Fig. 3b. Note that, within ±20 nm fabrication error that can be readily realized by the electron beam lithography (EBL), the amplitude and transmission phase are almost constant, and thus the influence of such fabrication error on imaging performance can be ignored.

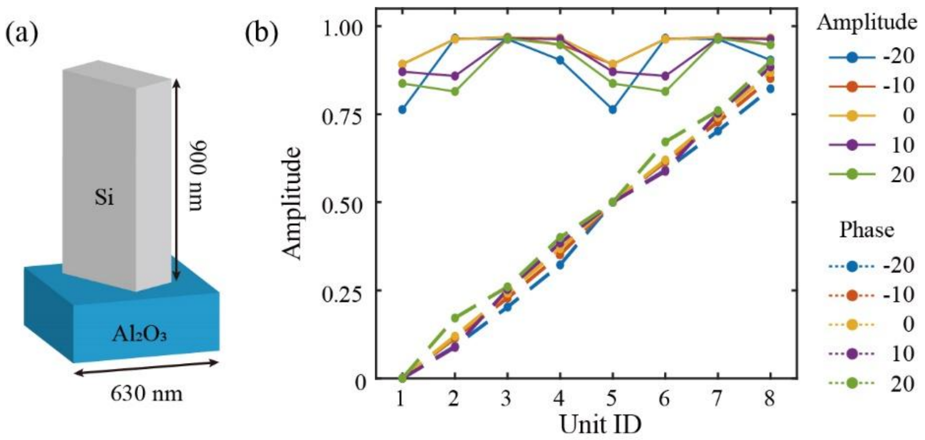

Supplementary Fig. 3. Unit cell design at the 1.55 µm wavelength. (a) Schematics of the unit cell. (b) Simulated polarization conversion amplitude and propagation phase of eight unit-cells at different fabrication errors.

Supplementary Table 5. Geometries of selected eight nanopillars at 1.55 µm wavelength and simulated polarization conversion amplitude.

	ID1	ID2	ID3	ID4	ID5	ID6	ID7	ID8
Width (nm)	550	495	460	520	255	230	205	145
Length (nm)	255	230	205	145	550	495	460	520
Amplitude	0.89	0.96	0.97	0.97	0.89	0.96	0.97	0.97

Supplementary Note 3: Vector coherent synthesis of two spin replicas at the image plane

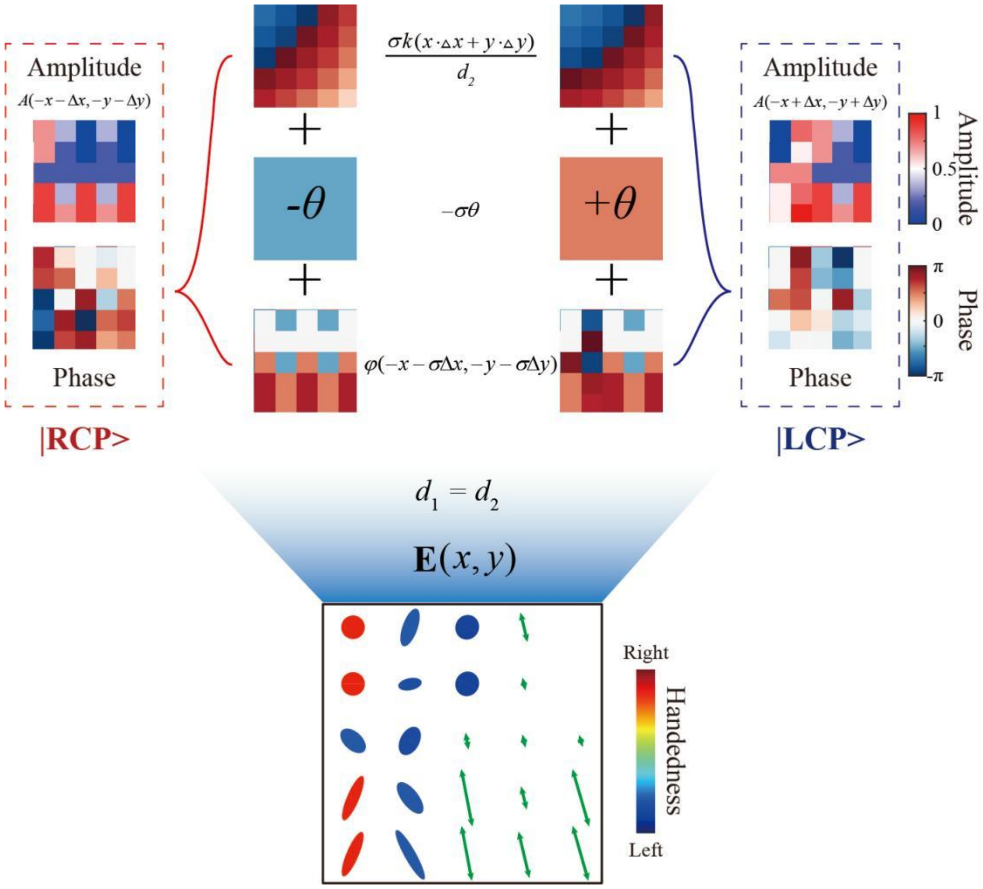

Supplementary Fig. 4. Vector coherent synthesis of two spin replicas at the image plane with spatial dislocation and overlap. Illustration of vector coherent synthesis of two spin replicas at the image plane within 5 × 5 pixels. The amplitude maps of LCP and RCP show a horizontal dislocation of 2 pixels. The differences between the phase maps are attributed to the different optical-path-determined propagation phase, the azimuthal angle of the incident polarization, and the inherent phase of the ciphertext. Since the complex-amplitude of the ciphertext and spin phase difference between the spin replicas are changed among the spatial pixels, the final polarization distribution is inhomogeneous and complex.

Supplementary Note 4: Design of the vector polarization analyzer

According to the theoretical and numerical analyses in the main text, a perfectly matched vector polarization analyzer is required at the front of sensors, whose spatial orientation γ fulfills:

$$\begin{aligned}\gamma &= \chi - \theta + \pi/2 \\ &= \frac{k(x\cdot\Delta x + y\cdot\Delta y)(d_1+d_2)}{2{d_2}^2} + \pi/2 \\ &= \frac{k(x\cdot\Delta x + y\cdot\Delta y)}{d_2} + \pi/2\end{aligned} \tag{6}$$

where Δx = 100 μm, Δy = 0, θ = 0, and $d_1 = d_2$ = 13 cm. The structural streamline of such a vector polarization analyzer belongs to the catenary of equal strength according to mathematical integral, as illustrated by the dash curves in Supplementary Fig. 5a, because its orientation profile linearly changes with the coordinates[22].

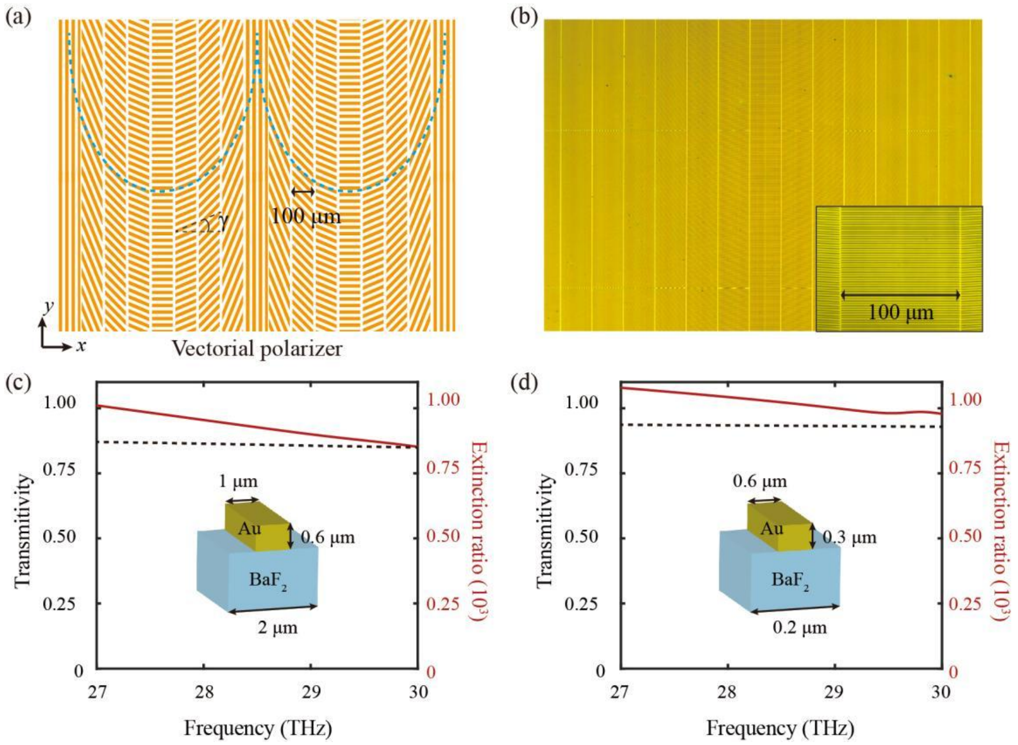

Supplementary Fig. 5. Vector polarization analyzer implemented by catenary-like gold gratings. (a) Schematic of the vectorial polarization analysis. (b) Optical microscope diagram of the fabricated sample. (c,d) Transmission and polarization extinction ratio of the gold gratings with different geometry parameters as a function of the operation frequency. Corresponding geometries are shown in the insets.

Since the pixel size of the optical steganographic ciphertext is 100 μm×100 μm, the period of the vector polarization analyzer that is composed of rotated gold (Au) gratings is correspondingly determined as 100 μm along the *x*-direction, as shown in Supplementary Fig. 5a. To obtain a high polarization detection efficiency, catenary-like gold gratings are utilized here, which can be taken as a series of periodic metallic stripes with space-variant orientation angles. Supplementary Fig. 5b presents a micrograph of the sample. Supplementary Fig. 5c shows the transmissivity and polarization extinction ratio of the gold grating as a function of the operating frequency. As can be seen from Supplementary Fig. 5d, by decreasing the period and height, the transmissivity (polarization extinction ratio) can be increased from 0.862 to 0.933 (from 938.8 to 1031.7) at the frequency of 28.28 THz (10.6 μm).

Note that, as revealed in Supplementary Eq. (6), the orientation profile of the vector polarization analyzer is linearly rotated with the *x*-axis. When it is misaligned along the *x* direction, one can rotate the incident polarization to compensate for the misalignment. In addition, the *y*-axis misalignment does not affect the imaging result, since the vector polarization analyzer is periodic along the *y*-axis.

Supplementary Note 5: Encrypt one confidential image into distinct ciphertexts

Supplementary Fig. 6 shows several ciphertexts (host images) that hid the same information, i.e., the letters "IOE", where the left and right panels show the results obtained by the normal and decryption cameras. As can be seen, these ciphertexts are entirely different because different random complex numbers are adopted as the encryption head (the first two columns shown in the image), as highlighted by the blue dashed box. The difference in the dark stripes of recovered "IOE" patterns demonstrates the indispensable role of two-column random complex numbers. These results indicate that the same information can be encrypted in various ciphertexts flexibly, but the same decryption camera can be used for all these ciphertexts. For real-world applications, such a one-to-many mapping relationship can confuse the eavesdropper, which helps to enhance the security level of the proposed vector visual cryptography.

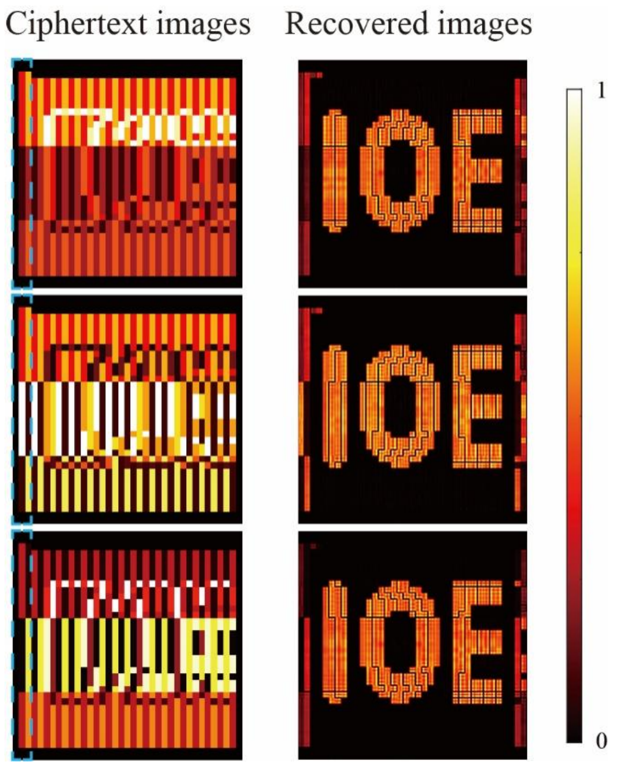

Supplementary Fig. 6. Encrypt the same confidential image into distinct ciphertexts. Left panel: different ciphertexts. As highlighted by the blue dashed box, the first two columns are random complex numbers that behave as the encryption head. Right panel: the corresponding recovered images through the proposed decryption camera.

Supplementary Note 6: Design of the complex-amplitude ciphertext

To design steganographic ciphertexts with arbitrary complex-amplitude distributions, the multiple meta-atom interference approach proposed in our previous work is employed, whose building block is a supercell consisting of 2×2 meta-atoms. As shown in Supplementary Fig. 7a, each meta-atom is square-shaped all-silicon nanofin, whose period and height are fixed at 4.55 μm and 6 μm. The diagonal nanofins have the same width, respectively impart propagation phase shifts of β_1 and β_2. According to the interference principle, to obtain arbitrary complex-amplitude distribution, the phases of the two diagonals should be written as:

$$\begin{aligned}\beta_1(x,y)&=\cos^{-1}[A(x,y)]+\varphi(x,y)\\ \beta_2(x,y)&=-\cos^{-1}[A(x,y)]+\varphi(x,y).\end{aligned} \qquad (7)$$

Supplementary Fig. 7b shows the phase shift of each nanofin as a function of the width, and then a look-table method is resorted to select the nanofin's width.

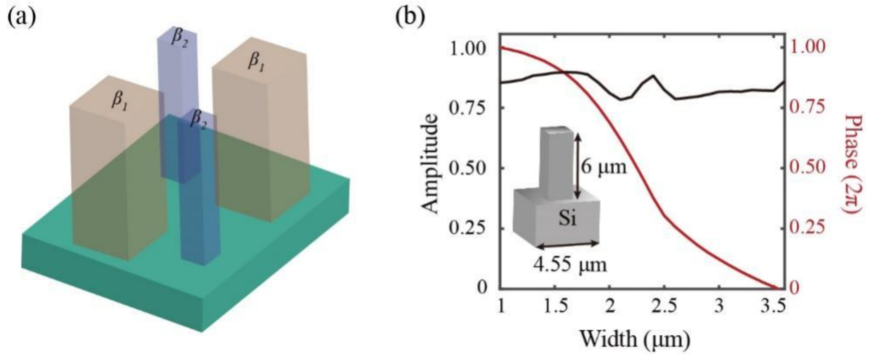

Supplementary Fig. 7. Optical complex-amplitude ciphertext implemented by multiple meta-atom interference. (a) Schematic of the building block, which is a supercell consisting of 2×2 meta-atoms. (b) The phase shift of each nanofin as a function of the width, with the period and height being respectively fixed as 4.55 μm and 6 μm.

Supplementary Note 7: Security verifications under circularly polarized incidences

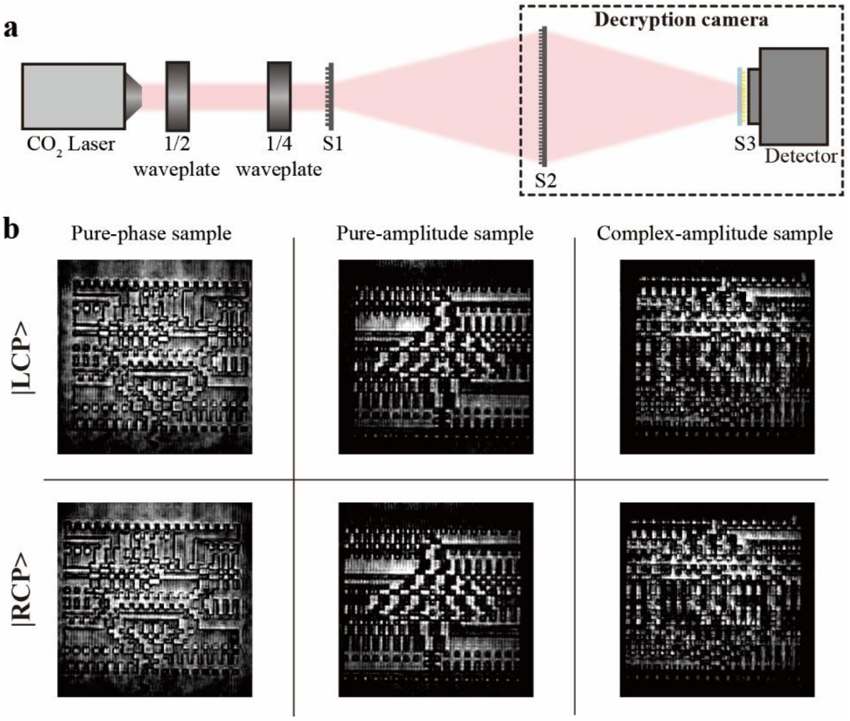

Supplementary Fig. 8. Spatial dislocation of images under circularly polarized incidence. (a) Schematic of optical setups. A pair of half-waveplate and quarter-waveplate were utilized to adjust the incidence polarization. (b) Measured imaging results for pure-phase sample, pure-amplitude, and complex-amplitude sample under LCP and RCP incidence, showing spatial dislocation. Note that, under circularly polarized incidence, the dual-axis metalens is equivalent to an ordinary single-axis lens.

References

1. Zhao, R. *et al.* Multichannel vectorial holographic display and encryption. *Light Sci. Appl.* **7**, 95 (2018).
2. Yue, F. *et al.* High-resolution grayscale image hidden in a laser beam. *Light: Science &Amp; Applications* **7**, 17129 (2018).
3. Deng, Z.-L. *et al.* Vectorial Compound Metapixels for Arbitrary Nonorthogonal Polarization Steganography. *Advanced Materials* **33**, 2103472 (2021).
4. Zhao, R. *et al.* Nanoscale Polarization Manipulation and Encryption Based on Dielectric Metasurfaces. *Advanced Optical Materials* **6**, 1800490 (2018).
5. Lim, K. T., Liu, H., Liu, Y. & Yang, J. K. Holographic Colour Prints for Enhanced Optical Security by Combined Phase and Amplitude Control. *Nature Communications* **10**, 25 (2019).
6. Song, Q. *et al.* Broadband decoupling of intensity and polarization with vectorial Fourier metasurfaces. *Nat Commun* **12**, 3631 (2021).
7. Li, J. *et al.* From Lingering to Rift: Metasurface Decoupling for Near- and Far-Field Functionalization. *Advanced Materials* **33**, 2007507 (2021).
8. Liu, H.-C. *et al.* Single-pixel computational ghost imaging with helicity-dependent metasurface hologram. *Sci Adv* **3**, e1701477 (2017).
9. Zheng, P. *et al.* Metasurface-based key for computational imaging encryption. *Science Advances* **7**, eabg0363 (2021).
10. Zheng, P. *et al.* Compressive Imaging Encryption with Secret Sharing Metasurfaces. *Advanced Optical Materials* **10**, 2200257 (2022).
11. Refregier, P. & Javidi, B. Optical image encryption based on input plane and Fourier planerandom encoding. *Opt. Lett.* **20**, 767–769 (1995).
12. Towghi, N., Javidi, B. & Luo, Z. Fully phase encrypted image processor. *J. Opt. Soc. Am. A, JOSAA* **16**, 1915–1927 (1999).
13. Matoba, O., Nomura, T., Perez-Cabre, E., Millan, M. S. & Javidi, B. Optical Techniques for Information Security. *Proceedings of the IEEE* **97**, 1128–1148 (2009).
14. Chen, W., Javidi, B. & Chen, X. Advances in optical security systems. *Adv. Opt. Photon., AOP* **6**, 120–155 (2014).
15. Yamamoto, H., Hayasaki, Y. & Nishida, N. Securing information display by use of visual cryptography. *Opt. Lett., OL* **28**, 1564–1566 (2003).
16. Yamamoto, H., Hayasaki, Y. & Nishida, N. Secure information display with limited viewing zone by use of multi-color visual cryptography. *Opt. Express, OE* **12**, 1258–1270 (2004).
17. Shi, Y. & Yang, X. Optical hiding with visual cryptography. *J. Opt.* **19**, 115703 (2017).
18. Ibrahim, D. R., Teh, J. S. & Abdullah, R. An overview of visual cryptography techniques. *Multimed Tools Appl* **80**, 31927–31952 (2021).
19. Guo, Y. *et al.* Merging geometric phase and plasmon retardation phase in continuously shaped metasurfaces for arbitrary orbital angular momentum generation. *ACS Photonics* **3**, 2022–2029 (2016).
20. Zhang, F., Pu, M., Luo, J., Yu, H. & Luo, X. Symmetry breaking of photonic spin-orbit interactions in metasurfaces. *Opto-Electron. Eng.* **44**, 319–325 (2017).

21. Balthasar Mueller, J. P., Rubin, N. A., Devlin, R. C., Groever, B. & Capasso, F. Metasurface Polarization Optics: Independent Phase Control of Arbitrary Orthogonal States of Polarization. *Phys. Rev. Lett.* **118**, 113901 (2017).

22. Pu, M. *et al.* Catenary optics for achromatic generation of perfect optical angular momentum. *Sci. Adv.* **1**, e1500396 (2015).
