# Supplementary material for: Meta-optics empowered vector visual cryptography for high security and rapid decryption
Source: Nat Commun. 2023 Apr 7;14:1946. doi: 10.1038/s41467-023-37510-z (PMC10081998; doi:10.1038/s41467-023-37510-z)
Supplement: Supplementary file 2 — Description of Additional Supplementary Files [file 41467_2023_37510_MOESM2_ESM.pdf]

Description of Additional Supplementary Files

Supplementary Movie 1: Real-time decryption properties of the meta- camera.
